# A Swarm of Bee Research

**DOI:** 10.1371/journal.pbio.2001736

**Published:** 2017-01-09

**Authors:** Lauren A. Richardson

**Affiliations:** Public Library of Science, San Francisco, California, United States of America

Bees are amazing little creatures; while some of them live solitary lifestyles, many bee species form large colonies, or hives, and function as a superorganism. Scientific interest in bees covers many different angles. Some researchers are interested in how bees learn and communicate as part of the superorganism. Others study how bees fly and recognize objects during flight—skills that have intriguing implications for development of manmade drones. And, as we are all sadly aware, there is intense research on bee pathogens and the contribution of those pathogens to colony collapse. In this Open Highlights article, I will discuss some of the recent advances in our understanding of these fascinating insects.

**Fig 1:**
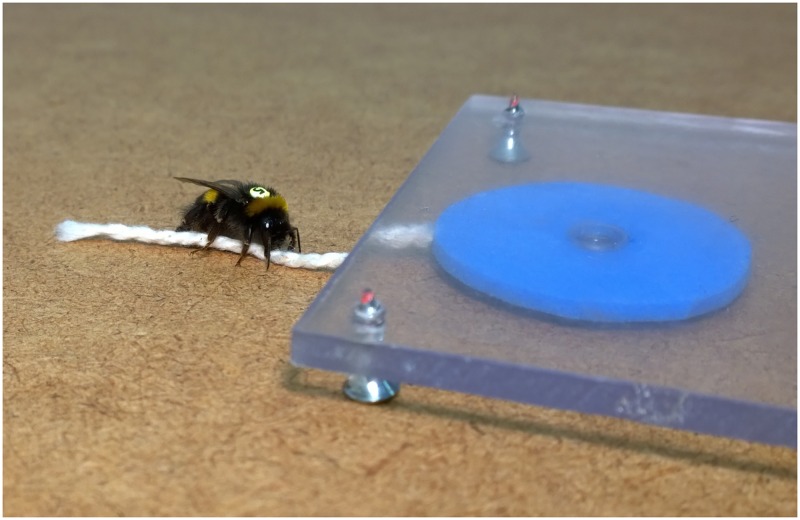
Bee culture? Bees trained to pull a string can teach this skill to other bees and disseminate this knowledge through the hive via cultural transmission. *Image credit*: *Olli Loukola*.

While one bee is small, a colony is mighty, and colonies are capable of extremely complex behaviors. How bees communicate and pass on knowledge has interested researchers for decades. In a recent paper in *PLOS Biology* [1], the authors demonstrate that bees are capable of social learning and cultural transmission—a first for invertebrates. In the study, the authors trained bees to perform a non-natural task: pulling a string to receive a sugar reward (Fig 1). The trained bees were then able to teach their colony-mates this string-pulling trick. Using a semi-natural colony simulation, they showed that the foraging bees learned the task, either from the trained bee or other learners (and very occasionally spontaneously), and that this behavior persisted even after the teacher was removed. This work demonstrates that bees can learn from watching other bees, and that this acquired skill can be spread and develop into a cultural element of the colony.

Bees can be trained in other non-natural tasks, and a *PLOS ONE* paper unearths new insights into learning and memory by developing an operant conditioning test whereby bees push a cap to uncover a food source [2]. Bee learning is also studied in more natural setting as well; the Asian species of honey bee *Apis cerana* is often attacked by hornets (*Vespa velutina*), and authors of another *PLOS ONE* paper [3] find that the presence of predator odor or alarm pheromone (a chemical signal secreted by bees as a warning to their conspecifics) compromises the bees’ ability to associate a stimulus with a sugar reward.

In addition to alarm pheromone, bees have evolved other mechanisms to warn their nest-mates of danger. In a second *PLOS Biology* article [4], the authors show that *Apis cerana* can not only warn nest-mates of a predator, but can tell them how big a threat the colony is facing. The ‘stop signal’ identified by the authors is a brief vibrational pulse that informs the nest of the level and context of the danger (is there a large hornet at the front door, or a small hornet at a distant food source?). Bees receiving these signals behave differently, either avoiding a food source, remaining in the nest, or attacking the invading hornet by forming a ball of bees that kills the attacker by heating it to death.

Communication within bee colonies is extremely complex. One of the primary mechanisms is through the famous “waggle dance,” which occurs on the dance floor as a means of signal amplification. But bees also engage in other types of collective behavior. In another *PLOS ONE* article, the authors provide evidence that Asian giant honeybees (*Apis dorsata*) engage in collective respiratory behavior to ventilate their hives for thermoregulation and freshness [5]. However, the collective, colony-benefiting behaviors of bees can also be exploited by others. A subspecies of the Western honeybee known as the Cape bee can invade a foreign colony, use its resources, and lay its own eggs, which are produced asexually and develop into female worker bees that are also able to reproduce in this asexual manner. The authors of a *PLOS Genetics* paper [6] compare the whole genome sequences of Cape bees with other honeybee subspecies to identify the mechanisms for this reproductive mode and social parasitism.

As mentioned above, another facet of bee research is into their mode of flying and information processing during flight. Authors of a *PLOS ONE* article [7] investigate how honeybees discriminate three-dimensional shapes; by training and observing free-flying bees, they show that bees can recognize objects by their three-dimensional shapes, and that they use specific combinations of flight maneuvers to extract depth cues, suggesting that bees have an optimized visuo-motor strategy for obtaining this information. In a *PLOS Computational Biology* paper [8], the authors build a model to understand the brain circuitry used to process environmental information during flight. Previous research has shown that bees estimate angular velocity from the speed that patterns move across their compound eyes, and this computational model was able to reproduce the departures from perfect behavior that are seen in real bees.

Another aspect of bee flight studied is how it changes due to pathogen infection. In this *Scientific Reports* article [9], the authors use radio frequency identification tags to track bees experimentally infected with the fungal pathogen *Nosema apis*. After four days of infection, the bees performed more flights of shorter duration, indicating a trade-off between foraging activities and the immune response. Another article published in *Scientific Reports* [10] also looked at how pathogens impacts bees’ behavioral performance and search strategy when introduced to a novel environment. These authors also find that *Nosema* infection caused short flights, but interestingly, the bees were still able to use the optimal search strategy, suggesting that search strategy is robust to physiological effects of pathogen infection. Related to these studies is one published in *Behavioral Ecology and Sociobiology* [11]. In this work, the authors infect honeybees with either Deformed wing virus or *Nosema* and investigated whether these infections altered the task allocation of bees within the colonies. Bees use an age-related division of labor, known as the temporal polyethism schedule. The authors found that the pathogens accelerated this schedule, but didn’t change the host behavioral repertoire, though they did see pathogen-specific changes in behavioral alterations.

Pathogens are one of the key stressors that are blamed for the colony collapse disorder plaguing regions like the United States. Since nearly one-third of the North American food supply is reliant on insects for pollination, the observed rise in colony collapse is highly concerning. However, it seems that a combination of stressful factors may be contributing to colony collapse. The authors of a *PLOS Pathogens* paper performed a large-scale controlled trial to determine how combinations of stressors impacted colony health [12], measuring bee pathogens, bee population, and weather conditions during one winter at three locations across the USA. While they note that one single harmful factor is not typically sufficient to trigger colony collapse, they do note the importance of *Varroa destructor*, a bee mite that not only steals nutrients from larvae but is also a vector for bee viruses. From data collected in this study, the authors build a predictive model to understand how the combination of stressors leads to colony collapse. Further studies on individual pathogens and their effects in combination will be needed to truly understand, and hopefully prevent, further loss of bee colonies.

For more detailed reading please see the associated PLOS Collection [13].
